# Psychological barriers to participation in the labor market: Evidence from rural Ghana^☆^

**DOI:** 10.1016/j.jdeveco.2026.103734

**Published:** 2026-02-12

**Authors:** Leandro Carvalho, Damien de Walque, Crick Lund, Heather Schofield, Vincent Somville, Jingyao Wei

**Affiliations:** a University of Southern California, United States of America; b Development Research Group, The World Bank, United States of America; c King’s College London, United Kingdom; d Cornell University, United States of America; e Department of Economics, NHH Norwegian School of Economics, Norway

**Keywords:** Mental health, Depression, Anxiety, Labor force participation, Work-from-home, Women, I15, J22, O15

## Abstract

Mental health conditions are strongly associated with reduced labor market participation, but the underlying channels through which such conditions impact labor supply remain unclear. We conduct a two-phase study decomposing this relationship by examining (i) job take-up decisions and (ii) labor supply, output, and earning conditional on job take-up, and (iii) quit rates. In Phase 1, women in rural Ghana are asked whether they would be willing to take-up a cash-for-work job during the lean season when alternative work is scarce. We find that individuals with depression and anxiety, which are common in this population, are much more likely to decline work offers outside the home but equally likely to accept work-from-home positions. In Phase 2, we randomly offer jobs at home to those who were willing to work from home, avoiding selection effects. Neither depression nor anxiety predict work completion, income, or quit rates when working from home. These findings suggest that poor mental health may harm labor market outcomes in traditional jobs outside of the home via reduced take-up, above and beyond the established negative impacts of mental health on productivity in work outside of the home. But, the results also suggest an alternative approach to improving labor market outcomes for those in poor mental health: work-from-home opportunities, which are not associated with lower take-up or lower productivity on the job for those in poor mental health.

## 1. Introduction

Poor mental health is prevalent in low-income populations around the world and has been associated with low labor supply and earnings, creating the potential for negative feedback loops which may perpetuate poverty (Ridley et al., 2020; World Health Organization, 2022b; Hakulinen et al., 2019; Biasi et al., forthcoming; Mojtabai et al., 2015; Lund et al., 2024; de Quidt and Haushofer, 2016; Barker et al., 2022; Fuhr et al., 2019; Patel et al., 2011; Weobong et al., 2017). While treating mental illness can have significant positive impacts on labor supply, these effects are not universal, with more muted or null results among some populations (Lund et al., 2024; Barker et al., 2022; Patel and Kleinman, 2003; Patel et al., 2017; Angelucci and Bennett, 2024; Baranov et al., 2020; Bhat et al., 2022).

These mixed results may have many potential drivers and relatively little is known about the channels through which poor mental health reduces labor supply and income. In order to better understand how mental health conditions affect these important outcomes and begin to develop policy solutions, we decompose the process and study three of its key components: (i) job take-up, (ii) labor supply, output, and earnings conditional on job take-up, and (iii) quit rates.

To accomplish this goal, we begin by measuring mental health in a low-income population of women in rural Ghana. We focus on women in a low-income setting as they suffer disproportionately from mental health conditions and have lower and more variable labor force participation (World Health Organization, 2022b). Consistent with global patterns, mental health in this population is poor, with 39.7% of individuals suffering from depression and 45.0% of individuals experiencing anxiety, relying on the “moderate” cutoffs for the corresponding mental health scales — the Patient Health Questionnaire (PHQ) and the General Anxiety Disorder scale (GAD) (Kroenke et al., 2001, 2003; Spitzer et al., 2006).

Next, to avoid selection due to search or endogenous job offers and isolate later portions of the causal chain, in Phase 1 of the study we make attractive and identical job offers to these individuals. The work offered is similar to cash-for-work opportunities often provided by governments with the aim of supporting the poor during the lean season in that it is part-time, low-skill, and offers a wage premium of roughly 50%. Further, the decision to work is high-stakes, with the income provided by the job accounting for about 46% of average total household income during this period. We elicit willingness to work under two different scenarios: work from home or work from a common worksite.^1^ This variation speaks to a key feature of depression and anxiety: those in poor mental health frequently seek to avoid new or potentially stressful situations. Allowing individuals to take-up work from a familiar environment may alleviate key stressors associated with labor force participation and increase take-up.

Our first key finding is that poor mental health (both anxiety and depression) is strongly associated with a reduced willingness to take up this attractive work opportunity. As can be seen in Fig. 2, participants who are anxious and depressed (i.e. scoring above the PHQ and GAD thresholds) are roughly 54% more likely to decline the offered work than those who are neither anxious nor depressed (11.9% vs. 7.7%). These differences are not due to existing work obligations, with more depressed individuals being less likely to cite existing work as a reason to decline and more likely to cite inability or unwillingness to take up the work as the reason for refusal. Nor do these differences in take-up appear to be driven by childcare responsibilities: respondents in poor and good mental health have similar numbers of young children, and results control for household demographics.

However, this strong association between depression and anxiety and the willingness to work is only present in work offered outside of the home; correlations between either aspect of mental health and willingness to work from home are quite small in magnitude and statistically insignificant (Table 5). This stark difference in take-up rates across work location for those in poor mental health is consistent with many of the symptoms of depression and anxiety – avoidance behaviors, low energy levels, or nervousness – which could make individuals less willing to leave the home despite the relatively generous wages.

These results suggest mental health is associated with reduced job take-up and low earnings in many contexts where work is primarily found outside of the home. This reduced willingness to take-up work outside of the home when in poor mental health may exacerbate known impacts of poor mental health on productivity at work in traditional (outside of the home) jobs (Ridley et al., 2020; Lund et al., 2024). This existing body of evidence raises the question of whether poor mental health may also impact labor outcomes in work from home by impacting performance on the job, even though individuals in poor mental health are equally likely to take up these jobs.

To answer these questions, we draw on Phase 2 of the study in which we enroll a random sample of women who are willing to work from home and randomly divide this sample into two groups: those who are offered to work from home (treated) and those who are not offered any work (control).^2^ The randomization also allows us to examine whether, conditional on being willing to take up a job working from home, this random job offer is equally valuable to depressed and anxious individuals. In other words, among those willing to take up the work, do those with poor mental health have similar labor supply, output, earnings, and quit rates?

To study these channels, we examine the labor supply, output, and earnings of participants who were randomly provided with jobs, including heterogeneity by baseline mental health which is, on average, quite poor in this population (34.9% and 32.8% of the sample experience at least moderate depression and anxiety at the baseline of Phase 2, respectively, based on standard cutoffs in the literature). Participants report bi-weekly on days worked and earnings in surveys. These data are combined with administrative data on work completed as a part of the study.

Unsurprisingly given that little work is available in the lean season, this random job offer greatly increases the likelihood of work among those offered the job, nearly quadrupling engagement with self-reported wage labor from 17% to 60% over the three months of work offered. We find that, although we cannot rule out small effects, in this group depressed and/or anxious individuals are just as likely to supply labor and produce as much as those in better mental health, and correspondingly earn similar amounts (Table 7). Further, poor mental health is associated with a small and marginally significant delay (improvement) in the time to quitting but does not predict quit rates overall.

In short, we conclude that depression and anxiety impede labor market success not only via lower productivity in traditional work contexts, but also because those with mental health conditions are much less likely to take up new labor opportunities. However, among individuals who take up home-based work – an outcome that is uncorrelated with baseline mental health – mental health does not reduce labor supply, output, or earnings, nor does it increase quit rates once they are on the job.

Beyond individual effects, we find suggestive evidence that poor mental health has the potential to create barriers to economic opportunity that extend to other household members. Participants with depression and anxiety were not only less likely to take up work opportunities themselves but also significantly less likely to indicate that other adult household members are available for work, both for work-from-home and for work-from-site opportunities. While not conclusive, these findings suggest that mental health challenges have the potential to create household-level constraints on labor market participation, potentially amplifying the economic consequences of poor mental health beyond the individual affected.

This paper contributes to three broad literatures. First, we contribute to research examining the relationship between mental health and labor market outcomes (Ridley et al., 2020; Hakulinen et al., 2019; Biasi et al., forthcoming; Lund et al., 2024). While prior work has documented that mental health interventions can improve labor supply and earnings in some contexts, we advance this literature in two ways. First, we decompose the channels through which mental health affects labor outcomes, which is important both to understanding the contexts in which this relationship will be stronger or weaker and to begin to consider appropriate policy responses. Second, we demonstrate that the association between poor mental health and reduced job take-up is context-dependent: while depression and anxiety strongly predict lower acceptance of outside-the-home work opportunities, these same mental health conditions show no association with willingness to accept work-from-home positions. Further, among those who accept workfrom-home positions, we find no evidence that mental health affects productivity, labor supply, or retention — contrasting with existing evidence on mental health’s effects in traditional workplace settings and opening the door to an alternative way to successfully engage those in poor mental health in the labor market.

Second, we contribute to the literature examining low labor force participation in low- and middle-income countries. While much of this work has focused on demand-side constraints such as job availability and search frictions, or supply-side factors like skills and transportation costs, we highlight a mechanism through which poor mental health may create additional barriers to labor force entry (Rossi, 2022; Collier et al., 2016; Goldberg and Reed, 2023; Carranza and McKenzie, 2024). Our evidence demonstrates that even when attractive job opportunities are directly offered, poor mental health – which is highly prevalent in this context – substantially reduces take-up in traditional employment opportunities outside of the home, suggesting an important but previously overlooked constraint on engagement with the labor force. Further, although only suggestive, our analysis of respondents’ decisions regarding household members’ willingness to work highlights the potential for the effects of poor mental health to extend beyond the impacted individual.

Finally, we inform the literature on cash-for-work programs, which are widely used as anti-poverty tools in low-income countries (Besley and Coate, 1992; Bertrand et al., 2021; Murgai et al., 2016; Imbert and Papp, 2015). These programs typically operate through standing job offers outside of the home, assuming that those most in need will self-select into the work. Our findings suggest that poor mental health – which is disproportionately prevalent among the poor – may limit engagement with these programs precisely among those they are intended to help. This has important implications for program design: screening for mental health conditions and providing complementary mental health interventions, or innovating to provide other targeted intervention designs such as work from home arrangements, may be necessary to achieve the full potential of cash-for-work as an anti-poverty tool.

The remainder of the paper begins with background on the context and study design in Section 2. We outline our conceptual framework in Section 3. The empirical approach is described in Section 4. Section 5 provides results while Section 6 concludes.

## 2. Context and study design

Our study is conducted in northern Ghana, in rural areas outside of the city of Tamale. The population is heavily agricultural and the poverty rate in this area is high, with over 70 percent of people defined as multi-dimensionally poor in the study districts (Ghana Statistical Service, 2023). Similar to many other areas with high poverty rates, mental health conditions are relatively high in the region: Ae-Ngibise et al. (2023) observe a prevalence of probable depression of 15.6%, and Barker et al. (2022) find that about 58% of the general population suffers from symptoms associated with some degree of psychological distress (compared to about 13 percent in the United States according to Dhingra et al., 2011).^3^

### Timing –

The study is conducted in the lean season, after planting and before harvest of the major staple grain, maize. The lean season offers limited work opportunities, especially opportunities for wage labor. Because many households are running low on grain and have limited income, this time is typically a period of relatively high stress. The study occurred in two phases over a period of five months (See Table 2 for a study timeline). Phase 1, which occurred in the first two months, examined the participants’ willingness to take up new work opportunities both at worksites and from home. Phase 2, which spanned three months following Phase 1, offered randomly selected individuals from the group willing to work from home the opportunity to work from home stitching bags as described below. Phase 2 was divided into six 2-week periods with data collection on mental health, labor supply and earnings collected in face-to-face interviews at baseline and endline (following period 6), and via phone surveys at the end of each period in-between, resulting in 5 total phone surveys.

### Work opportunity –

We offer to randomly selected participants in Phase 2 the opportunity to stitch bags – allowing us to observe output each period – at their home. Prior to beginning their work, participants are trained over 3 days by professionals to ensure they are able to meet the minimum quality standards. Materials for the bags are dropped off at the beginning of each two-week period and the finished bags are picked up three times throughout the period. Payment is piece-rate (GHS 12 per completed bag) and made immediately upon the pickup of the finished product. The time required depends on the amount of work offered each period, but is on average about 3 to 4 days of work per period and ranges from 1.5 to 6 days per two-week period.

The jobs offered are an important potential source of income in this context. Because work opportunities are often limited during the lean season, the average household income at the beginning of Phase 2 is GHS 474 (USD 32.7) per month, equivalent to GHS 237 per 2-weeks period. The majority of this income typically comes from male adults, who earn more per hour and work outside of the home more frequently. Participants are offered an average of GHS 108 (around 7.5 USD) each two-week period. The job offers have the potential to increase total household income by 45.6% on average given the low labor demand outside of the study. However, it is also important to note that, as is typical during lean seasons, consumption is significantly higher than income during this time such that the job offer is roughly equivalent to 14% of household consumption. The hourly wage premium is designed to be similar to many cash-for-work programs such as India’s Mahatma Gandhi National Rural Employment Guarantee Scheme (NREGA), and is roughly 50% higher than the prevailing day labor rate for women (Nagaraj et al., 2016).^4^

The fact that we offer the same jobs uniformly to all participants allows us to address some of the potential areas of concern around confounding. Specifically, this approach allows us to: (1) abstract away from differences in search behavior which could generate selection into who is observed, and (2) hold constant the features of the job which might otherwise endogenously (and unobservably) vary with the individual’s characteristics such as their mental health status.

### Phase 1 –

This first phase starts with the recruitment of participants in 30 rural communities with limited work opportunities during the lean season. The communities are distinct villages with 115 households on average. In each community, we target households using a random walk procedure. To be eligible, households must have access to a phone in order to respond to phone surveys – though nearly all did – and have five or fewer adults and a total household size of less than 16 members.^5^ The random sampling in the targeted communities results in 838 eligible households. However, because Phase 2 was limited to women, we also limit Phase 1 analysis to women for consistency. Of the 838 eligible households, 514 have female respondents.^6^ Twenty-three of the female respondents are individually ineligible due to age (<18 or >65) and are not asked to provide information about their willingness to work. The remaining 491 eligible respondents are considered in our “willingness-to-work sample”, used to assess the relationship between a respondent’s mental health and their willingness to take-up new job offers.

In each household the respondent is then asked to provide basic socio-demographic information about the household. They are then read a description of the work opportunity and asked if they would be willing to do such work, both for work from home and work outside of the home.^7^ We randomize whether the respondent is asked first about work from home or work from a worksite, and control for an order dummy in all specifications despite the fact that there is no significant impact of the order in which the questions were asked. Respondents are aware that not all individuals would be offered the work, but that it would only be offered to those interested in taking it up. Next, respondents are asked to report whether other adults in their household would be interested in the job or not, again responding both for work from home and for work outside of the home. Finally, participants also respond to the PHQ-2 screening tool, assessing depression, and the GAD-2 module, assessing anxiety. The screening criteria, data collection, and sample sizes are summarized in Table 1.

### Phase 2 –

Study participants in this phase are first sampled through the process described for Phase 1 participants. To be eligible, individuals must be non-pregnant women aged 18–65 years who are willing to work from home. In Phase 1, they either indicated their willingness to work directly (N = 491), or another individual in the household indicated whether they would be willing to work (N = 1076).^8^ Among these 1567 women, 1447 are willing to work from home. To select participants for Phase 2, we first selected households with at least one eligible woman who was willing to work and then randomly sampled 400 of these households. Among the sampled households, if there was more than one eligible woman willing to work, one woman per household was randomly selected to be enrolled. We were unable to recontact 10 of these individuals because they had left their village. Because they were unaware of their assigned arm prior to dropping from the study we omit them. The remaining 390 completed an informed consent and were enrolled, constituting the final study sample for Phase 2.

Following the random selection, we return to the households to conduct a baseline survey with the Phase 2 participants. The baseline survey includes measures of labor supply, income, consumption, and mental health (PHQ-8, GAD-7, Cohen’s Perceived Stress Scale, and the Penn State Worry Questionnaire). Additionally, the participants are asked to confirm again their willingness to work, which all did. The 390 individuals completing this baseline survey are considered our “interested sample”.^9^ Then, of the 390 “interested” participants, we randomly make job offers to work from home to 320 of these participants (our treated group) and not to the other 70 participants (our control group).^10^

Both treated and control participants in Phase 2 are interviewed by phone every two weeks during the study. Both groups also have a final in-person interview (endline survey) once the work is completed. During the bi-weekly phone interviews we elicit labor supply, income, and consumption measures as well as the participant’s mental health (GAD2 and PHQ-2). Additionally, treated participants have administrative data collected on their completion of bags each period, noting both the number and quality as well as the payment made for the work. The endline interview includes the same measures of labor supply, income and consumption, and the mental health measures used in the baseline. Attrition over the course of Phase 2 was minimal: only six participants did not complete the endline survey (3 in each arm, see Table A.1).

### Measurement –

Mental health is proxied by depression, assessed using the Patient Health Questionnaire (PHQ), and anxiety, assessed using the General Anxiety Disorder Questionnaire (GAD) (Kroenke et al., 2001, 2003). Both the PHQ and the GAD are among the most frequently used scales to screen for depression and anxiety globally, and have been validated and used previously in Ghana (Adjorlolo, 2020; Anum et al., 2019; Barthel et al., 2014; Weobong et al., 2009). We use the 8-item version of the PHQ and the 7-item version of the GAD at the baseline and endline in Phase 2, and the 2-item version of the PHQ and GAD in phone surveys and Phase 1.^11^ The local language has no colloquial written form. Hence, to ensure consistent administration of the items and fidelity to the scales in this context we discussed appropriate approaches with a local expert, Dr. Ben Weobong. The final protocol included translating each question in the survey into the local language, back translation (via a recording) from a second individual blind to the original scale, editing for consistency as required, and then generating a final recording of the item. The enumerator explained the scale, played the recording of each item one by one, and recorded the participant’s responses.^12^

The main outcomes of interest are: the women’s willingness to work (Phase 1), and their labor supply, output, earnings, and quit rates (Phase 2). Willingness to work, both for the respondent and other household members, is captured in Phase 1 as explained above. Participants in the “interested sample” in Phase 2 report their labor supply and earnings over the past ten days in all surveys. They are also asked to report the labor supply and earnings of other household members over the age of 12 for the same period. Not all participants include their study earnings in their reported earnings. In the main tables, we correct the reported earnings to always include the study earnings. The study work is organized in periods of two weeks and the survey corresponding to each period is administered in the last four days of the two week period. The ten-day recall therefore allows us to capture labor and income during that period only and does not cover previous periods.^13^

### Partnerships –

The study is implemented in partnership with Innovations for Poverty Action (IPA) Ghana, who collect the data, and Presbyterian Agricultural Services (PAS), who manage the hiring and work-related tasks (in a fashion similar to Banerjee et al., 2020).

### Safety procedures –

Given that we assess participants’ mental health status, we implemented safeguards for participants showing severe mental distress. We establish a referral system in partnership with the Mind ‘N’ Health Foundation that provides phone counseling services in the local languages. The support services are available free of charge to the participants. The services are presented sensitively as “a person to talk with about any concerns that are on your mind” to address potential stigma around mental health. Mind ‘N’ Health’s providers include psychologists, psychology therapists, certified counselors, and counseling experts. Additional referral procedures to physical facilities are in place in case of concerns around self-harm, though these were not needed during the study.

### Summary statistics

#### Phase 1.

**Fig. 1.**
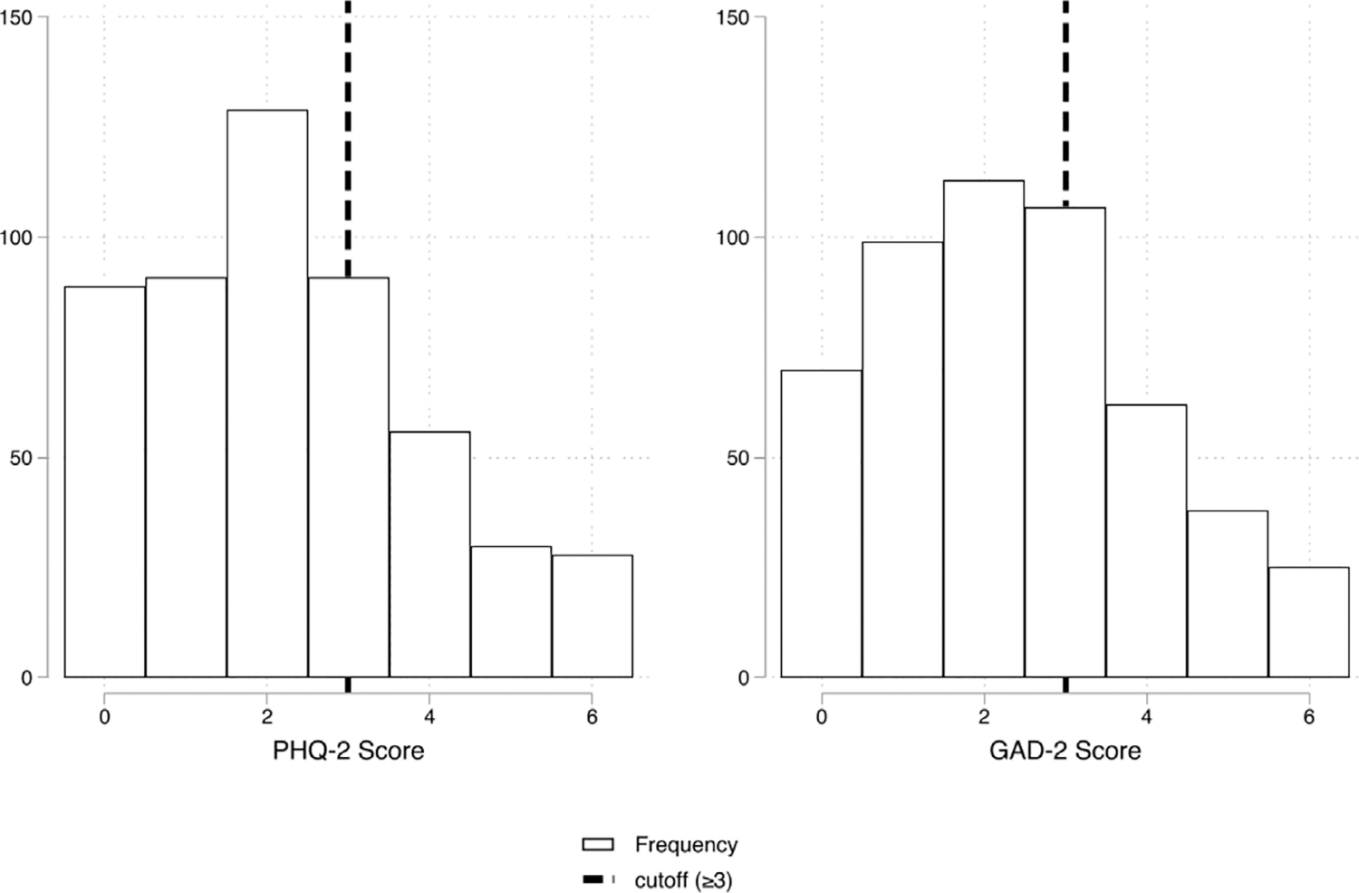
Distribution of mental health scores among female participants. *Note:* The figure shows the distribution of depression scores (PHQ-2, left) and anxiety scores (GAD-2, right) of the Phase 1 sample. The vertical dashed lines indicate clinical cutoffs (≥3), above which scores are considered to indicate elevated symptoms of depression or anxiety.

As noted earlier, mental health is relatively poor in this population: Fig. 1 plots the distribution of depression and anxiety scores among women in Phase 1. Approximately 40% of the women are at or above the standard cutoff for further screening for depression, and 45% are at or above the standard cutoff for further screening for anxiety.

Table 3 reports basic summary statistics describing the Phase 1 sample. Column 1 provides the mean (standard deviation) and Columns 2 and 3 provide the coefficient of a simple ordinary least squares estimation between each variable shown in the table and a binary variable indicating whether the participant’s PHQ-2 (depression) and GAD-2 (anxiety) score are above the clinical thresholds, as well as the associated standard errors.

Households in this area are large, with an average of eight members. The average household earns about GHS 560 per month (equivalent to 37 USD at the time of the survey), with earnings winsorized at the 95th percentile. The participants are 36.7 years old on average and 74% of them report some paid or unpaid work (excluding domestic labor) in the past 12 months, the majority being self-employed or working for the family business.

The PHQ-2 – where lower scores indicate better mental health – has a negative but insignificant correlation with the size of the household and household income as well as associations with engagement with specific types of labor (agriculture and work in a household enterprise). The GAD-2 shows similar correlations with specific types of jobs. In addition, women who are heads of household experience more anxiety, while both being married and being the spouse of the head is associated with better mental health.

#### Phase 2.

In Table 4, we report summary statistics describing our Phase 2 sample at baseline, separately for the treated and control groups. Column 3 provides a *p*-value for a test of equality of means between the two groups. Treatment and Control are similar on the all baseline covariates tested, suggesting the randomization was successful.

## 3. Conceptual framework

We next discuss how poor mental health – particularly depression and anxiety – may influence job acceptance and on-the-job performance. Although our data do not separately identify the mechanisms at work, the goal is to provide a conceptual framework for what may underlie these relationships. Depression is characterized by low mood, loss of interest or pleasure (anhedonia), fatigue, and reduced motivation, whereas anxiety is marked by excessive fear, negative rumination, avoidance, and safety-seeking behaviors (World Health Organization, 2022a). Although each condition has distinct symptomatology, they are highly co-morbid.

Workers’ willingness to accept a job – our question of interest in Phase 1 – reflects the perceived benefits and costs of taking the job. On the benefit side, anhedonia may lower utility from tasks that would otherwise be enjoyable and reduce utility derived from wage-financed consumption (Cléry-Melin et al., 2011; Pizzagalli, 2014). At the same time, low mood, fatigue, and reduced motivation may increase the disutility of work effort, reducing take-up (Treadway et al., 2012). Anxiety may also create negative anticipatory utility about potential adverse events (Caplin and Leahy, 2001), with increasing selective attention towards threatening stimuli (Armstrong and Olatunji, 2012). For example, worry about being able to perform the job proficiently can impose psychological costs even before work begins. Moreover, if individuals anticipate that poor mental health will reduce their productivity and earnings, they may be less likely to accept a job offer, with pessimism further amplifying these concerns by causing them to overestimate the impact of mental health on work (Abramson et al., 2024).

Beyond general impacts on take-up, both depression and anxiety are likely to affect the perceived benefits and costs of each job, leading to a stronger preference for home-based employment. The avoidance behavior and anhedonia common in anxiety and depression create barriers to engaging with opportunities outside familiar environments (Struijs et al., 2017). A central distinction between the two work settings is social exposure: outside work involves frequent interpersonal contact and crowding, which raises psychological costs for those with social anxiety (Ridley et al., 2020), and close supervision and evaluation can be taxing for individuals with anxiety or low self-esteem (Zhang et al., 2022). Stigma may compound these burdens, as workers with poor mental health may fear judgment, discrimination, or being perceived as less competent in face-to-face environments. Working from home also offers greater flexibility in pacing and scheduling—useful when mood and energy fluctuate or sleep timing is irregular (Jones et al., 1987). By contrast, on-site jobs impose fixed-time routines – preparing to leave, commuting, and managing meals away from home – that may raise perceived costs. In addition, rigid on-site schedules make juggling chores and caregiving more costly, especially under circumstances of depression and anxiety.

Finally, mental health may also affect on-the-job performance (de Oliveira et al., 2023) —our question of interest in Phase 2. Conditional on employment, deficits in attention, working memory, and executive control, together with sleep–wake dysregulation, low mood, fatigue, and reduced motivation, may raise the disutility of work effort and impair productivity (Burt et al., 1995; Snyder, 2013; Palacios-Barrios and Hanson, 2019; Semkovska et al., 2019; Airaksinen et al., 2005). Experimental work also shows that individuals with major depression do not increase physical effort in response to higher monetary rewards (Cléry-Melin et al., 2011; Treadway et al., 2012). These effects are likely to be especially pronounced for tasks that demand sustained concentration or complex problem-solving. Anxiety, in particular, is associated with fearfulness and negative rumination, which can impair performance (Eysenck et al., 2007; Abramson et al., 2024).

Empirically, it is important to acknowledge that observed associations between mental health and job take-up and on-the-job performance observed may not be causal. They may also reflect omitted variable bias. For example, poverty elevates the risk of common mental disorders (Ridley et al., 2020) and may directly impede employment and productivity (Kaur et al., 2025). We therefore adjust for observable confounders in our analysis, while acknowledging that residual confounding may remain.

## 4. Empirical approach

### 4.1. Phase 1 – Mental health and willingness to work

As the first step in the chain of employment, we begin by examining women’s willingness to work when facing new opportunities. We start by investigating whether anxiety and depression predict one’s willingness to work, keeping in mind that all the participants are offered the same simple jobs with a relatively high pay, and that they are asked for their willingness to work in two scenarios: either at a nearby workplace or from home.

We estimate the following equation:

(1) $$Y_{i}=\alpha_{0}+\alpha_{1}\text{Mentel}\;{\text{health}}_{i}+X_{i}\alpha_{2}+\epsilon_{i,}$$

where *Y*_*i*_ is a binary variable equal to one if individual *i* is willing to work and to zero otherwise. We estimate Eq. (1) separately for the willingness to work from home and for the willingness to work from a worksite. “Mental Health” is one of the two mental health variables measured in Phase 1 —PHQ-2 or GAD-2— standardized for ease of interpretation, or an average of the two standardized variables. ***X*** is a vector of controls including the order of the willingness to work questions (which was randomized), whether the household head or his spouse were both present or not (which influenced the selection of the respondent, as explained in Section 2), the household size and number of adults, age of the respondent, and marital status. We select these controls because they are unlikely to be influenced heavily by the participant’s mental health status. We report the values of *α*_1_ for different measures of mental health in Table 5. Panel A presents results about work from a local worksite decisions and Panel B presents results about working from home. All regressions use robust standard errors.

To assess the robustness of our estimates, and to reduce the risk of omitted variable bias, we present the results of the estimation with alternative measures of mental health in Appendix Tables. Additional details of these robustness checks are provided in the results section.

### 4.2. Phase 2 – Mental health and the effects of job offers

In the second phase, we randomize job offers among the participants who are willing to work from home. We estimate the effects of the job offers on labor supply, output, earnings, and quit rates as well as whether mental health mediates the effects of these job offers. Specifically, we estimate:

(2) $$Y_{i,t}=\gamma_{0}+\gamma_{1\;}\text{Job}\;{\text{offer}}_{i}+\gamma_{2\;}\text{Mentel}\;{\text{health}}_{i,0}+\gamma_{3\;}\text{Mentel}\;{\text{health}}_{i,0}*{\text{Job}\;{\text{offer}}_{i}+T_{t}+X}_{i}\gamma_{4}+\zeta i,t,$$

where *Y*_*i,t*_ is outcome *Y* for individual *i* in period *t*, “Job offer” is equal to one if *i* was randomly offered a job, “Mental Health” is one of the mental health variables measured in period 0 (the baseline), and T_*t*_ are period fixed effects. ***X*** controls for the same demographics as in Eq. (1), as well as controls for which individual in the household responded in Phase 1, but omits the controls for order effects and presence of another household member as they are not relevant in Phase 2. When we estimate Eq. (2), we cluster standard errors at the individual level given the multiple periods. As with the Phase 1 analysis, we also examine the robustness of these estimates to a number of variants such as binary measures of mental health, use of the short form measures of mental health, and changes to the control set.

## 5. Results

### 5.1. Phase 1: Mental health and willingness to work

**Fig. 2.**
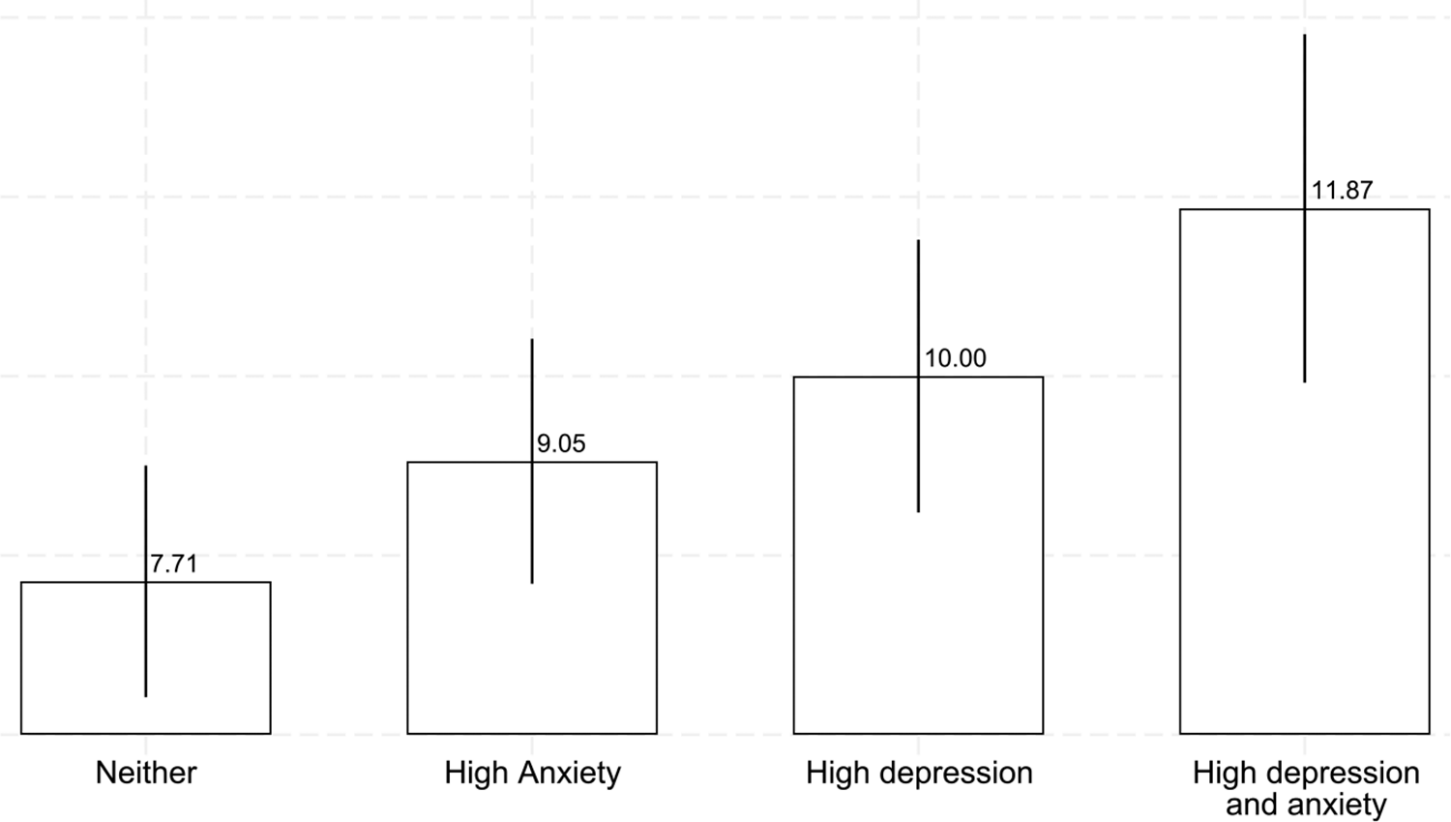
Mental health and work offer refusal rates. *Note:* The bar heights show the percentage of participants who refuse the work offer, pooling across questions about work from home and work outside of the home (N = 982). The spikes correspond to the standard errors of the means. High anxiety corresponds to a GAD-2 score of three or more. High depression corresponds to a PHQ-2 score of three or more.

Both anxiety and depression are strong predictors of the willingness to work pooling across locations, with marked differences in refusal rates among those with greater anxiety, greater depression, or both, compared with individuals in good mental health (Fig. 2). Specifically, refusal rates are 1.3 (2.9) percentage points or 17.4 (29.7)% higher among participants with high anxiety (depression). Being identified with both high depression and high anxiety is associated with refusal rates that are 4.16 percentage points or 54.0% higher.

In Table 5, we test the statistical significance of these associations, this time distinguishing between working outside of the home (top panel) or from home (bottom panel). We find that the PHQ-2 and the GAD-2 strongly correlate with the decision to accept a job offer if the work must be done at a worksite, but not if it can be done from home. In our main specification (column 1), on average, an increase of one standard deviation on the PHQ-2 scale (1.68 points) is associated with a reduction of 3.0 percentage points in the likelihood of accepting the offer at a worksite. The association with the GAD-2 (SD = 1.64 points) is of a similar magnitude. The association between the index of mental health and willingness to take up work is slightly larger in magnitude, 3.7 percentage points, but not significantly different than the individual measures of mental health.

These results are in strong contrast to the associations between mental health and willingness to take up work from home. The point estimates when considering work from home are small in magnitude – none larger than 1.2 percentage points –, positive, and non-significant. These correlations suggest that the relationship between mental health and the take-up of employment opportunities may vary substantially with the context in which the work is provided, and may exacerbate effects in low income settings where remote work is less common.

To provide additional confidence in these results, next we also include additional controls: the participant’s relationship to the household head, her type of work, the number of young and older children in the household and the household total food expenditures (column 2). The coefficients remain quiet stable with this change. Additionally, in columns 3 to 7, we expand further upon the potential set of controls by shifting to the baseline data from Phase 2. While this approach allows us to include additional controls only available in the baseline survey, it limits the sample. Specifically, we can only include those who were both the individuals who were the respondents willing to work from home in Phase 1 and who were randomly selected to participate in Phase 2.^14^ Column 3 replicates the estimates from column 1, but using the Phase 2 overlapping subsample. Column 4 adds variables related to economic well-being and preferences — the participant’s years of education, sector of past employment, total value of the household’s assets, an index of household wealth (measured by the EquityTools index, Chakraborty et al. (2016)), time (measured by the Consideration of Future Consequences Scale, Strathman et al. (1994)) and risk preferences (general willingness to take risks, Dohmen et al. (2011)). Column 5 includes controls for the occurrence of recent shocks – death of a relative, loss of livestock, other accident, job loss, natural disasters, religious events – in addition to expenses related to the shocks, and a measure of social support influencing one’s ability to cope (the ‘Multidimensional Scale Of Perceived Social Support’, Zimet et al., 1988). Column 6 includes controls for nutritional status —an index of food security,^15^ a standardized household dietary diversity score (FAO, 2013), and the participant’s body weight. Column 7 includes all controls from columns 3 to 6 in a single regression. Again, the negative association between mental health measures and willingness to work from home remains stable or increases in absolute value with these additional controls while the associations between mental health and work from home remain small and insignificant.

We conclude from this exercise that the estimated correlation between mental health and willingness to work is very stable, even when omitting nearly all controls or controlling for a large number of potentially confounding factors. This finding reinforces our confidence in the Phase 1 results.

#### 5.1.1. Robustness

In order to further assess the robustness of our estimates we provide the results of several additional analyses. In Table A.2, instead of the continuous measures of mental health, we use binary indicators equal to one if the respondent’s score on the PHQ-2 and GAD-2 screening tools exceeds the clinical threshold. In Table A.3 we remove the demographic controls (the age of the respondent, their marital status and the number of adults and number of household members in the household) included in Table 5.

#### 5.1.2. Why are individuals unwilling to work?

To further understand these associations, we asked participants who were not willing to work why they did not want to take up the job. In Table 6, we correlate the mental health measures with the reasons given for refusing the work offer. Each cell presents the coefficient from a distinct regression, pooling across work locations. Notably, people with higher depression and anxiety scores are significantly less likely to say that they already have paid work as a reason for refusing job offers. Much of this difference appears to be accounted for by a positive association between poor mental health and being unable or unwilling to work, though these associations are less precise and the relationship is only statistically significant at the 10% level for depression. The correlations between mental health and other reasons such as a lack of transportation or student status are generally weaker and not statistically significant.

The evidence that the lack of take-up is not driven by better work opportunities is also bolstered by the incomes of the various groups during Phase 2. As can be seen in Fig. A.2, those offered a job in the study immediately earn roughly 100 GHS per period, while both those who were interested but not offered the job and those who were not interested in taking up the work have average incomes of approximately 10 GHS per period and are statistically indistinguishable. These differences remain relatively constant over time. If refusals were driven by other better labor opportunities we would expect the “not interested” group to have either a higher level of income or a stronger upward trend in income. As neither of these conditions is met, these relationships provide additional evidence that those who opted not to take up the offered work did not do so due to other better labor opportunities.

#### 5.1.3. Summary

In short, we find that both depression and anxiety are strongly predictive of turning down a lucrative job opportunity when that opportunity is outside of the home, but are not associated with any differences in the desire to take-up work in the home. The differences in engagement with work outside of the home are large, with refusal rates roughly twice as high among those who are both anxious and depressed compared to those who are neither anxious nor depressed. Further, the lack of engagement with work offered outside of the home is not consistent with patterns we would expect if better work opportunities were available elsewhere. Taken together, these results suggest that a lack of willingness to engage with work that is typically available in low-income contexts – work outside of the home – has the potential be an important driver of the gap in labor market outcomes between those in good and poor mental health, but that work-from-home opportunities have the potential to be particularly valuable to those in poor mental health. Finally, in Appendix B, we find that these associations are not limited to the respondent themselves; respondents in poor mental health are also less likely to report that others in the household would be willing to take up the work. It is difficult to fully parse the channels that drive this relationship. While only exploratory and suggestive given the potential confounds, these findings could suggest that mental health conditions may have the potential to extend beyond individual labor supply decisions to household-level economic opportunities.

### 5.2. Phase 2: Mental health and behavior on the job

Next, we examine the relationship between mental health and job performance. Participants are offered jobs to be performed from home, avoiding selection concerns since mental health affects willingness to work outside the home but not willingness to work from home. To accomplish this goal, we randomly assign individuals – regardless of mental health status – to treatment (offered a job) and control (not offered a job) groups. We survey both groups every two weeks. We estimate the intent-to-treat effect of job offers on labor market outcomes and examine whether depression and anxiety moderate these effects.

#### 5.2.1. Job performance

**Fig. A.1.**
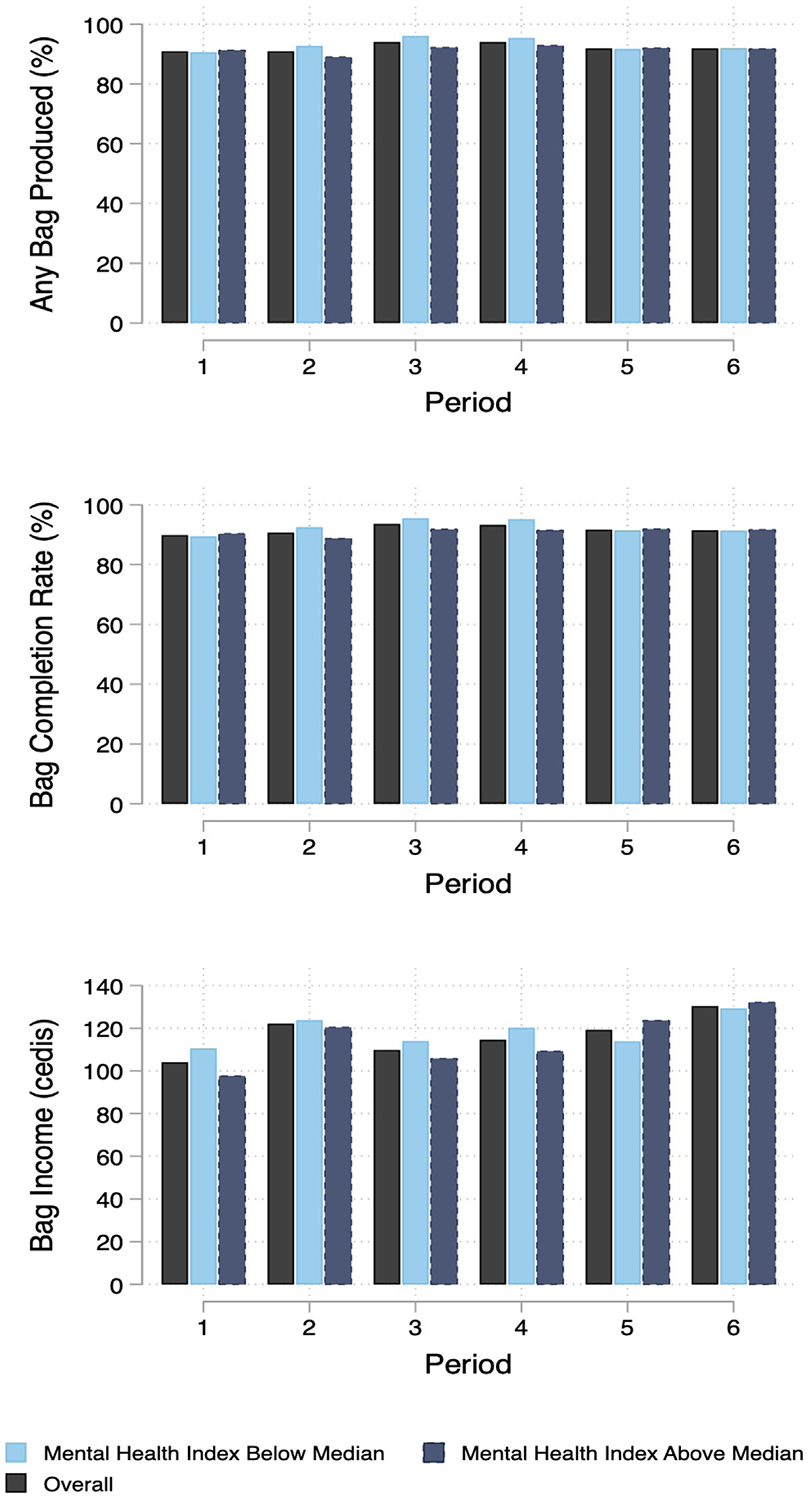
Take up of work. *Note:* This figure provides data on engagement with the work opportunity over time among those offered a job in Phase 2. The top panel shows the fraction of workers producing at least one bag during the period. The middle panel presents the fraction of the offered bags that were completed. The bottom panel shows the income from the bags in Ghanaian Cedis (GHS). Within each figure and each study period, the black bar shows the overall average, the light blue bar shows the average for individuals with below average mental health, and the dark blue bar shows the average for individuals with above average mental health.

**Fig. A.2.**
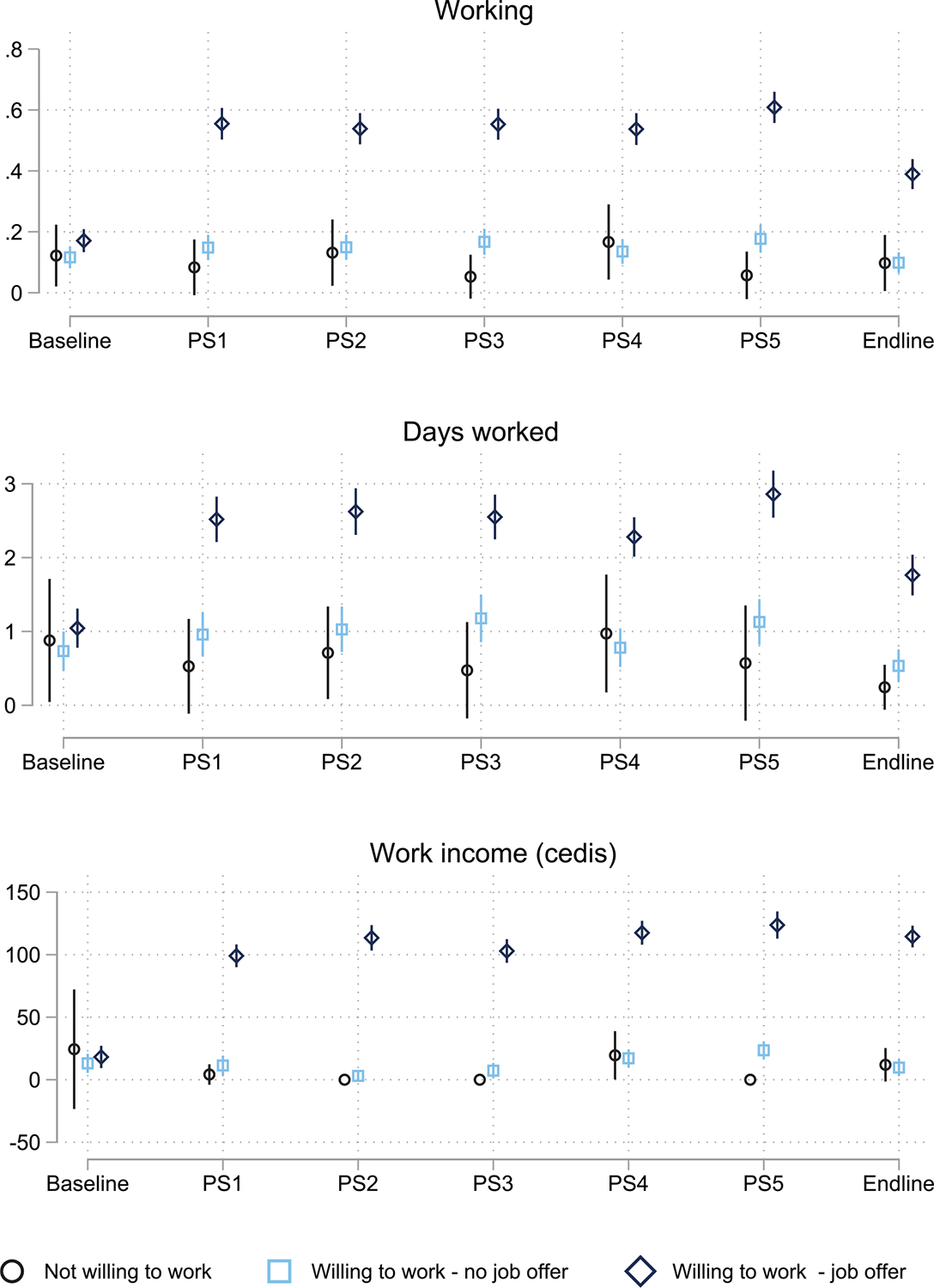
Job offer and labor supply. *Note:* The top figure shows the mean fraction of people working over the past 10 days(and 95 percent confidence intervals around the means) over time by period, for three groups of participants: (1) those who are not willing to work, (2) those who are willing to work but do not receive a job offer, and (3) those who are willing to work and receive an offer. The center panel shows the mean number of days worked and 95 percent confidence intervals around the means for the same three groups. The bottom panel shows the means (and 95 percent confidence intervals around the means) of earnings (GHS, on the vertical axis).

Similar to many other studies which offer work during the leanseason or at a wage premium, take-up of the offered work is high (Bessone et al., 2021; Breza et al., 2018, 2021; Kaur et al., 2025). As shown in Fig. A.1, the overall bag completion rate exceeded 90%, with 93.1% of individuals submitting at least one bag in any given period, on average. Fig. A.2 further shows large and persistent gaps in the extensive margin (any work at all), the intensive margin (days worked), and earnings between those offered a job and those not offered one, providing a strong “first stage”.

Table 7 presents the results of estimating Eq. (2), allowing us to estimate differences in performance at work among those with better and worse mental health. Panel A examines work status using a binary variable for whether the individual reported working in that period, either as a part of the study or outside of it. Panel B examines self-reported days worked, assigning a zero for individuals who did not work that period. Panel C examines output, measured as the number of bags produced — this analysis is restricted to participants who were offered a job. Panel D studies labor income, again assigning a zero for individuals who reported no work during the period, either as a part of the study or outside of it.

We begin by estimating the overall impact of a job offer on each of these outcomes in Column 1, findings large effects on work engagement, number of days of worked, and earnings, as noted earlier.^16^ We then turn to our primary question of interest: whether the impact of a job offer varies by mental health status. The mental health status measures are standardized baseline measures of depression (Col. 2), anxiety (Col. 3), and an average of the two standardized measures (Col. 4). The interaction term in Eq. (2) allows us to test whether the participants’ baseline mental health moderates the effect of the job offers.

The interaction terms are relatively moderate in magnitude and are never statistically significant. Take for example the effect on the extensive margin shown in the top panel. The coefficient on the interaction term is −0.036, which represents roughly 8.3% of the main effect of being offered a job. Similarly, for earnings, the coefficient on the interaction term in column 4 is −5.38 GHS compared to a mean increase of 102.72 GHS when offered a job. Turning to bags produced, we find no evidence that poorer baseline mental health is associated with lower productivity, with coefficients that are both positive and negative and never statistically significant. We cannot reject the hypothesis that productivity is similar across levels of mental health. This similarity in outcomes across those in good and poor mental health is also visually evident in Fig. A.1.^17^ However, the limited sample size also means we cannot rule out modest effects on these outcomes. Results are similar when we use instead binary measures for mental health (Table A.4), short-form measures of mental health (PHQ-2 and GAD-2, Table A.5), or when demographic controls are omitted (Table A.6).

#### 5.2.2. Quit rates

Finally, to examine the relationship between mental health and work retention, Table 8 reports correlations between mental health indicators and quit rates among those offered a job stitching bags. We use two measures of quitting: (i) non-submission of bags in the final period and (ii) a variable indicating the last period in which the participant submitted any bags, which captures the timing of quitting. About 8% of participants did not submit bags in the final period. Consistent with our earlier findings on mental health and job performance, we find no significant relationship between baseline mental health and this measure of quitting. When looking at the timing of quitting, the associations with mental health are also small and statistically insignificant. For example, a one-standard-deviation increase in anxiety is associated with persisting 0.09 periods longer, but this estimate is imprecise.

## 6. Conclusion and discussion

This study decomposes the relationship between mental health and labor market outcomes into distinct components: willingness to engage in work, output and labor supply on the job, and retention. Our findings reveal that depression and anxiety are significant predictors of labor supply through reduced willingness to accept work opportunities when the work requires to leave home, though not when the work is offered at home. Further, in contrast to the existing literature on work in traditional (away from home) environments (Ridley et al., 2020; Lund et al., 2024), we find no evidence that mental health influences productivity or retention among those who choose to work and can do so from their home.

These results have important implications for both theory and policy. First, they suggest that a potential mechanism through which poor mental health may reduce labor market engagement is by creating psychological barriers to participation, particularly in contexts that amplify avoidance behaviors and other psychological changes associated with poor mental health. This finding may help to explain mixed results in the literature regarding the impact of mental health treatments on labor outcomes. While studies often measure labor outcomes through productivity, earnings, or days worked, our results suggest focusing on willingness to work is also essential to capturing the full effects of mental health improvements.

Second, our findings have implications for the design of anti-poverty programs, particularly cash-for-work initiatives common in low-income settings. The job offered in this study resembles many such programs in both wage and timing: compensation was roughly 50% above prevailing market rates, and work was offered during a period of limited alternative employment opportunities (Nagaraj et al., 2016). The fact that mental health significantly correlates with job take-up suggests that integrating mental health support services into existing workfare programs could increase their effectiveness. Further, because mental health is associated with job take-up when the job must be done outside, but not when one can work from home, this indicates that more flexible work arrangements can be a promising way of including people with poor mental —who often represent a substantial share of the population in low-income settings. As the output associated with many existing workfare programs is already limited, altering production tasks for home-based work may not have large productivity costs. Further, the ever increasing reach of internet connectivity may continue to expand opportunities for productive work from home opportunities even for those with limited education. For example, workers could undertake data labeling tasks to train AI or engage in tasks with both personal and community benefit that could be verified via phone photographs (e.g. digging a drainage ditch in front of their home with before and after photos). Moreover, given that poor mental health appears to create barriers to accepting work opportunities even when little other work is available, policymakers may need to reconsider the assumption that those most in need will naturally self-select into these programs.

However, a work-from-home approach is not without tradeoffs beyond the added complexity. For example, traditional work outside of the home might offer additional benefits through social interaction and network building that could improve mental health outcomes. But emphasizing such arrangements could create equity concerns: some individuals might benefit from increased social interaction while others with mental health conditions would be excluded entirely, missing both potential social benefits and economic opportunities. In short, this research suggests the potential for work-from-home arrangements to benefit those in poor mental health is high, but more research is also needed to fully flesh out both logistical challenges and potential trade-offs.

Finally, our results point to important directions for future research. While we document that mental health is strongly associated with willingness to work outside home, understanding the specific psychological mechanisms behind this reluctance – whether related to self-efficacy, misperception, or other factors – remains an important area for investigation. Additionally, examining whether similar patterns hold in other contexts, with other types of work, piece rate vs. wage work, or lower or higher-paying work would help establish the generalizability of these findings. Key features of depression and anxiety are not only behavioral (avoidance of aversive stimuli and social withdrawal), but also mood-related (sadness, anhedonia and fearfulness) and cognitive (negative views of self and the future, time related preferences such as discounting future rewards for more immediate rewards) (Haushofer and Fehr, 2014). Our study suggests opportunities for future research to explore the cognitive and mood-related correlates of avoidant behavior and how these may serve to maintain psychological cycles of poverty. For example, future studies might explore whether take-up of employment opportunities among people living with depression or anxiety is associated with improvements in mood and cognition; and whether these changes precede or follow changes in earnings.

## Figures and Tables

**Table 1:** Screening process and survey administration details.

	Screening criteria	Data collected - household	Data collected - individual	Sample size
Phase 1, screening survey	18–65 years old Maximum 15 household members and 5 adults Access to cellphone	Household roster Housing characteristics Economic standing (short)	Willingness to work (self and others) Mental health (PHQ-2, GAD-2) Miscellaneous	491 female respondents
Phase 2, baseline & endline	Phase 1 criteria Female Not pregnant Willing to work from home (either self or other-reported during Phase 1)	Household roster Consumption and expenditures (long) Savings, loans, gifts shocks, property (long)	Mental health (PHQ-8, GAD-7, PSWQ, CPSS) Labor supply Time & risk preference Health Life satisfaction (long)	390
Phase 2, 5 rounds of phone surveys	Identical to Phase 2 baseline and endline	Household roster (updates) Consumption and expenditures (short) Savings, loans, gifts, property (short)	Mental health (PHQ-2, GAD-2) Labor supply Life satisfaction (short)	Average 92.5% response rate. See Table A.1 for balance by round

*Note:* To select participants for Phase 2, we first selected households with at least one eligible woman who was willing to work. We then randomly selected 400 households. Among these households, if there was more than one eligible woman willing to work, one woman per household was randomly selected to be enrolled. We were able to locate 390 of the women selected for consent and enrollment. The remaining 10 had left the village, but were unaware of their treatment assignment at the time of leaving and are excluded from the study. The content of the surveys is listed in the order it was asked.

**Table 2:** Study timeline.

Phase 1 survey	Phase 2 baseline	Intervention, Phone surveys	Phase 2 endline
March-April 2024	April-May 2024	June-August 2024	August-September 2024

*Note:* This table details the timeline of the study.

**Table 3:** Summary statistics – Phase 1.

	(1)	(2)	(3)
	Mean	Corr. with PHQ-2	Corr. with GAD-2
Household characteristics			
Number of adults	3.63	−0.01	−0.01
	(1.315)	(0.045)	(0.045)
Number of members	8.02	−0.08	−0.06
	(2.993)	(0.045)	(0.045)
Income (30 days)	558.88	−0.06	−0.00
	(540.930)	(0.046)	(0.046)
Respondent characteristics			
Age	36.66	0.00	−0.03
	(10.881)	(0.045)	(0.045)
Married	0.89	−0.07	−0.11*
	(0.316)	(0.045)	(0.045)
Head of household	0.04	0.08	0.17***
	(0.193)	(0.045)	(0.045)
Spouse of head	0.79	−0.08	−0.17***
	(0.410)	(0.045)	(0.045)
Other relation to head	0.18	0.04	0.09*
	(0.380)	(0.045)	(0.045)
Employed (12 months)	0.74	−0.05	−0.07
	(0.442)	(0.045)	(0.045)
Works in own/HH firm	0.55	−0.13**	−0.17***
	(0.498)	(0.045)	(0.045)
Works in agriculture	0.21	0.13**	0.12**
	(0.409)	(0.045)	(0.045)
Employed for wages	0.04	0.04	0.13**
	(0.203)	(0.045)	(0.045)
High PHQ-2	0.40		
	(0.490)		
High GAD-2	0.45		
	(0.498)		
Observations	491	491	491

*Note:* This table presents summary statistics for Phase 1 of the study and their correlations with mental health measures (PHQ-2 and GAD-2). Standard deviations are in parentheses in Column (1), and standard errors are in parentheses in Columns (2) and (3). Income is reported for the past month, in GHS and winsorized at the 95th percentile. “Employed (12 months)” indicates employment status in the past 12 months. “Works in own/HH firm” indicates self-employment or employment in a household enterprise. “Works in Agriculture” indicates primary employment in agricultural activities. “Employed for Wages” indicates salaried employment or daily labor for cash. “Head of household”, “spouse of head”, and “other relation to head” are mutually exclusive categories indicating the respondent’s relationship to the household head. “High PHQ-2” and “High GAD-2” indicate PHQ-2 and GAD-2 scores above their respective clinical thresholds (Kroenke et al., 2003, 2007). Correlations in Columns (2) and (3) are calculated using standardized PHQ-2 and GAD-2, respectively.

Statistical significance at the 0.10, 0.05, and 0.01 levels is indicated by *, **, and ***.

**Table 4:** Summary statistics – Phase 2.

	(1)	(2)	(3)
	Treatment	Control	p-value: (1)==(2)
Household characteristics			
Number of adults	3.63	3.63	0.998
	(1.33)	(1.21)	
Number of members	8.27	8.30	0.943
	(3.07)	(2.53)	
Income (10 days)	150.07	194.63	0.247
	(286.29)	(313.18)	
Respondent characteristics			
Age	37.27	38.27	0.502
	(11.37)	(10.99)	
Married	0.89	0.83	0.175
	(0.32)	(0.38)	
Years of education	1.15	1.19	0.931
	(2.84)	(2.95)	
Employed (10 days)	0.17	0.17	0.993
	(0.38)	(0.38)	
Works in agriculture	0.14	0.10	0.335
	(0.35)	(0.30)	
Employed for wages	0.01	0.01	0.713
	(0.10)	(0.12)	
Other Employment	0.14	0.13	0.844
	(0.34)	(0.34)	
High PHQ-8	0.37	0.33	0.527
	(0.48)	(0.47)	
High GAD-7	0.28	0.24	0.516
	(0.45)	(0.43)	
Observations	320	70	390

*Note:* This table presents summary statistics for Phase 2 of the study, comparing treatment and control groups across household and respondent characteristics. Column (1) shows the mean and standard deviation (in parentheses) for the treatment group, while column (2) shows the same statistics for the control group. Column (3) presents p-values from tests of equality between treatment and control means. “Income” denotes reported income from working in the past 10 days. “Employed (10 days)” indicates employment status in the past 10 days. “Works in agriculture” indicates primary employment in agricultural activities. “Employed for Wages” indicates salaried employment or daily labor for cash. “Other Employment” denotes work outside of agriculture, salaried work or daily labor (such as self-employment or work in a household firm). “High PHQ-8” and “High GAD-7” indicate scores above their respective clinical thresholds (Kroenke et al., 2009; Spitzer et al., 2006).

**Table 5:** Correlation between mental health and willingness to work.

	(1)	(2)	(3)	(4)	(5)	(6)	(7)
	Willingness to work outside the home						
PHQ-2 (std)	−0.030*	−0.030*	−0.035	−0.050*	−0.038	−0.039	−0.055*
	(0.017)	(0.018)	(0.024)	(0.028)	(0.024)	(0.024)	(0.029)
GAD-2 (std)	−0.029*	−0.025	−0.054**	−0.063**	−0.055**	−0.058**	−0.071***
	(0.015)	(0.016)	(0.022)	(0.025)	(0.024)	(0.025)	(0.027)
Ave. of PHQ-2 (std) and GAD-2 (std)	−0.037**	−0.035*	−0.053**	−0.069**	−0.056**	−0.059**	−0.077**
	(0.019)	(0.020)	(0.025)	(0.030)	(0.026)	(0.027)	(0.031)
Dep. Var. Mean	0.89	0.89	0.91	0.91	0.91	0.91	0.91
	Willingness to work from home						
PHQ-2 (std)	0.004	0.003	0.008	0.010	0.007	0.008	0.008
	(0.012)	(0.012)	(0.008)	(0.010)	(0.007)	(0.008)	(0.008)
GAD-2 (std)	0.011	0.012	0.005	0.005	0.005	0.006	0.003
	(0.012)	(0.012)	(0.005)	(0.005)	(0.005)	(0.006)	(0.004)
Ave. of PHQ-2 (std) and GAD-2 (std)	0.010	0.009	0.008	0.010	0.007	0.009	0.007
	(0.014)	(0.014)	(0.008)	(0.010)	(0.007)	(0.009)	(0.008)
Dep. Var. Mean	0.95	0.95	0.99	0.99	0.99	0.99	0.99
Observations	491	482	156	156	156	156	156

*Note:* This table examines the relationship between mental health indicators and willingness to work in two settings: working outside and working from home for eligible female respondents. “Willingness to work from home” and “Willingness to work outside” are binary indicators for whether the respondent accepted the respective work offers. PHQ-2 (std) and GAD-2 (std) refer to standardized scores from the Patient Health Questionnaire-2 and Generalized Anxiety Disorder-2 screening tools. The “Ave. of PHQ-2 (std) and GAD-2 (std)” takes the mean of these two standardized variables. All specifications include controls for whether the work from site question was asked first (the order was randomized), whether both the household head and spouse were present, the age of the respondent, their marital status and the number of adults and number of household members in the household at the time of the survey. In addition, column 2 includes controls for the participant’s relationship to the household head, her type of work, the number of young and older children in the household and the household total food expenditures. Column 3 replicates the estimates from column 1, but using the Phase 2 overlapping subsample. Column 4 includes controls related to economic well-being and preferences: the participant’s years of education, years of education for the households most educated member, sector of past employment for the participant and household members, total value of the household’s assets, an index of household wealth, time and risk preferences. Column 5 includes binary variables that capture the occurrence of recent shocks, total expenses due to these shocks, and a measure of social support influencing one’s ability to cope. Column 6 controls for measures of nutritional status: an index of food security, an index of dietary diversity and the participant’s body weight. Column 7 includes all of the controls from columns 3 to 6 in a single regression. The p-value in the last row tests the equality of coefficients between Work from Home and Work from Site specifications within each column. Robust standard errors are in parentheses.

Statistical significance at the 0.10, 0.05, and 0.01 levels is indicated by *, **, and ***.

**Table 6:** Association between depression and reasons for work refusal.

	(1)	(2)	(3)	(4)	(5)
	Have work	Household work	Unable or unwilling	Student	Transportation
PHQ-2 (std)	−0.087**	0.008	0.089*	−0.023	0.023
	(0.033)	(0.044)	(0.048)	(0.020)	(0.040)
GAD-2 (std)	−0.094**	0.090	0.051	−0.013	−0.008
	(0.039)	(0.055)	(0.061)	(0.016)	(0.036)
Ave. of PHQ-2 (std) and GAD-2 (std)	−0.102**	0.050	0.082	−0.021	0.012
	(0.039)	(0.053)	(0.058)	(0.020)	(0.043)
Reason share	0.114	0.380	0.367	0.0253	0.115
Observations	79	79	79	79	52

*Note:* This table examines how a respondent’s mental health relates to their stated reasons for why they would not accept work. Each cell contains the coefficient from a distinct regression, pooling across work locations. The sample for Columns 1 to 4 is restricted to respondents who refused offers to work from home or from a worksite. The sample for Column 5 is restricted to respondents who refused to work from a worksite only. PHQ-2 (std) and GAD-2 (std) refer to standardized scores from the Patient Health Questionnaire-2 and Generalized Anxiety Disorder-2 screening tools. The “Ave. of PHQ-2 (std) and GAD-2 (std)” takes the mean of these two standardized variables. Each column represents a different reason for refusing work: (1) already having paid work, (2) household work responsibilities including taking care of children, (3) being unable or unwilling to work including sickness, old age, and unwilling to work, (4) being a student, and (5) transportation difficulties (only relevant to work outside). Reason Share indicates the proportion of respondents who cited each reason for refusing work. All specifications include controls for whether the work from site question was asked first (the order was randomized), whether both the household head and spouse were present, the age of the respondent, their marital status and the number of adults and number of household members in the household at the time of the survey. Standard errors, clustered at the individual level, are in parentheses.

Statistical significance at the 0.10, 0.05, and 0.01 levels is indicated by *, **, and ***.

**Table 7:** Work offers, labor supply, income and mental health.

	(1)	(2)	(3)	(4)
	–	PHQ-8 (std)	GAD-7 (std)	Ave. of PHQ-8 (std) and GAD-7 (std)
Working				
Job offer	0.435***	0.432***	0.429***	0.429***
	(0.032)	(0.032)	(0.033)	(0.032)
Baseline mental health measure		0.041	0.036	0.044
		(0.026)	(0.026)	(0.027)
Baseline mental health measure × job offer		−0.036	−0.015	−0.028
		(0.031)	(0.030)	(0.032)
Control Mean	0.172	0.172	0.172	0.172
Observations	2188	2188	2188	2188
Days worked				
Job offer	1.693***	1.678***	1.662***	1.666***
	(0.204)	(0.205)	(0.207)	(0.206)
Baseline mental health measure		0.177	0.205	0.219
		(0.191)	(0.176)	(0.198)
Baseline mental health measure × job offer		−0.141	−0.117	−0.144
		(0.211)	(0.193)	(0.219)
Control Mean	1.052	1.052	1.052	1.052
Observations	2188	2188	2188	2188
Bags produced				
Baseline mental health measure		−0.032	0.171	0.084
		(0.175)	(0.163)	(0.187)
Mean		8.998	8.998	8.998
Observations		1920	1920	1920
Work income				
Job offer	103.232***	103.040***	102.543***	102.714***
	(3.559)	(3.602)	(3.710)	(3.664)
Baseline mental health measure		4.956	5.112	5.752
		(3.934)	(4.063)	(4.357)
Baseline mental health measure × job offer		−5.671	−3.782	−5.378
		(4.394)	(4.449)	(4.830)
Control Mean	14.97	14.97	14.97	14.97
Observations	2188	2188	2188	2188

*Note:* This table examines the relationship between mental health and labor market outcomes in the Phase 2 sample of 390 participants across 6 rounds of post-baseline data collection. PHQ-8 (std) and GAD-7 (std) refer to standardized scores from the Patient Health Questionnaire-8 and Generalized Anxiety Disorder-7 screening tools. The “Ave. of PHQ-8 (std) and GAD-7 (std)” takes the mean of these two standardized variables. The table presents four panels: Working (binary indicator), Days Worked (count of days), Bags produced (count of bags in each period), and Work Income (combines study and non-study income, in local currency units, winsorized at the 95th percentile). “Job offer” is a binary indicator for whether the respondent is randomly offered a job. All regressions include period fixed effects, controls to indicate whether the Phase 2 participant was also the Phase 1 respondent or was referred by a female respondent (leaving being referred by a male as the omitted group), as well as basic demographic controls outlined in Section 4: age and marital status of the respondent, household size and number of adults in the household. The standard errors, clustered at the household level, are in parentheses.

Statistical significance at the 0.10, 0.05, and 0.01 levels is indicated by *, **, and ***.

**Table 8:** Association between baseline mental health and quit rate.

	(1)	(2)	(3)
Did not turn in bags at endline			
PHQ-8 (std)	0.013		
	(0.017)		
GAD-7 (std)		−0.002	
		(0.017)	
Ave. of PHQ-8 (std) and GAD-7 (std)			0.007
			(0.019)
Observations	320	320	320
Dep. Var. Mean	0.0813	0.0813	0.0813
Last period in which participant submits bags			
PHQ-8 (std)	−0.021		
	(0.084)		
GAD-7 (std)		0.090	
		(0.076)	
Ave. of PHQ-8 (std) and GAD-7 (std)			0.042
			(0.089)
Observations	320	320	320
Dep. Var. Mean	5.60	5.60	5.60

*Note:* This table examines the relationship between baseline mental health measures and job retention/quitting behavior among participants that are offered the work. PHQ-8 (std) and GAD-7 (std) refer to standardized scores from the Patient Health Questionnaire-8 and Generalized Anxiety Disorder-7 screening tools. The “Ave. of PHQ-8 (std) and GAD-7 (std)” takes the mean of these two standardized variables. In the top panel, the outcome is a binary variable indicating if a participant quit before the endline. The outcome in the bottom panel is the number of periods a participant worked before quitting. The standard errors, clustered at the individual worker level, are in parentheses.

Statistical significance at the 0.10, 0.05, and 0.01 levels is indicated by *, **, and ***.

## Data Availability

The data and code are available at the Harvard Dataverse (https://doi.org/10.7910/DVN/WJ2O4R).

## Footnotes

1 The location of the worksite was either in the participant’s village or a nearby village and participants were responsible for their own transportation, which is often by foot.

2 We did not offer jobs outside of the home both for logistical reasons and because those taking up the jobs are likely to be highly selected given the prior results. Further, as individuals cannot and should not be compelled to work, examining outcomes conditional on willingness to work is the more policy-relevant and ethical approach.

3 Ae-Ngibise et al. (2023) define “probable depression” as the combination of a PHQ-9 score greater than four and reporting at least two cardinal symptoms of depression or anhedonia (loss of interest or pleasure). Barker et al. (2022) construct an index based on three measures: the Kessler Psychological Distress Scale, a self-rating of mental health taken from the Behavioral Risk Factor Surveillance System (BRFSS) and a self-report of days in the month without poor mental health.

4 The wage premium depends on the assumed number of hours required per bag, estimated by the stitching trainers, and ranges from 20% to 80% depending on the assumptions used.

5 The household size was set to ensure that we could survey the complete households in a reasonable time, and because in the second phase, we aimed to offer jobs that would substantially improve the economic situation of the participating households. This criteria screened out 366 households (30.3%) of the 1202 households originally contacted.

6 If both the household head and his spouse are present, one of them is randomly selected to be the main respondent. If either the head or his spouse are absent, the enumerator randomly selects one of the present adult household members to respond. This sampling procedure was used as this was a pilot study for a future RCT and we were considering adding men to the full RCT the following year.

7 The job offer is described as: “We are conducting this part of the survey to identify individuals who might be willing to participate in the study on people’s economic circumstances during the lean season. You have been randomly selected to answer this part of the survey about yourself and other household members. If a member from your household is selected to participant in the study, the person will be given a gift for subsequent surveys they answer. In addition, some of the study participants will be offered work for a period of 12 weeks; others will not be offered any work. Study participants who are offered work will work stitching bags. Each bag will be paid 12 GHS. No previous experience is required. We will provide training. Note: if your household is selected, only one member from your household will be selected to participate”. This portion of the survey was prior to, but separated from, the mental health measures by other content (e.g. questions regarding gender norms).

8 500 of the individuals for whom another individual responded were women and 576 were men.

9 In a given household, the participant from Phase 1 is not necessarily the same person as the “interested” participant from Phase 2. They overlap in 156 cases. This is because the Phase 2 participant was randomly selected among all eligible women from Phase 1 who signaled interest in the job, whether they were the Phase 1 respondent herself or not.

10 The experiment in phase 2 was a pilot study for a larger experiment to be conducted the following year studying the effects of income volatility and uncertainty. Following the anticipated design for the full study, treated participants were all offered the same type of work (stitching bags from home). However, expected workload (and hence income) and forward-looking knowledge of that workload over time varied across arms: one group had the same income in every period, the second group had variable income across time that was known in advance, and the last group had both variable and uncertain income. These differences were not described in the brief description of the work during Phase 1 and hence could not have impacted take-up decisions. No participants declined to participate in Phase 2 after learning these details during the informed consent. Because the differences in the timing of the workload are not relevant to the questions of interest in this paper and the results are statistically indistinguishable across treated arms, we aggregate these groups together in our analyses resulting in a treatment group that is substantially larger than the control. Analysis of the pilot data was not pre-registered. The patterns reported here emerged during exploratory analyses conducted to inform the design of the full study.

11 We follow established clinical thresholds for interpreting these scales. For the PHQ-2 and GAD-2, scores of 3 or higher (out of 6) indicate probable depression and anxiety respectively (Kroenke et al., 2003; Plummer et al., 2016). For the PHQ-8, we use the threshold of 10 or higher to identify potential depression (Kroenke et al., 2009). For the GAD-7, scores of 10 or higher indicate moderate anxiety requiring clinical attention (Spitzer et al., 2006).

12 Although use of recordings is not standard practice for these scales, the accuracy and reliability of the administration combined with strong comprehension in earlier pilots suggested they were the optimal approach for the local context.

13 While the 10-day recall omits 4 days of the period, the timing of the survey is random across treated and control participants, such that “missing” days are balanced.

14 Recall some individuals selected for Phase 2 were not Phase 1 respondents. Rather they were indicated as willing to work by another individual in their home.

15 Anderson index constructed from respondents response to food security questions.

16 Bag completion data in Fig. A.1 is administrative but the work data in Table 7 is self-reported. Notably, this results in a gap between reported levels of work in the two datasets. We believe some women did not classify bagstitching as “work” since it occurred at home and outside their usual activities. However, to be conservative, we retain self-reported work status in Table 7.

17 We also explore the possibility that effects may vary with the work schedule the participant was assigned to (stable, predictable volatility, or unpredictable volatility) but find no differences.

## Appendix A. Additional figures and tables

See Figs. A.1 and A.2, Tables A.1–A.6.

**Table A.1:** Response rates of phone surveys and endline.

	Survey response rate		
	Control	Treatment	Difference
Period 1	0.900	0.925	−0.025
Period 2	0.957	0.938	0.020
Period 3	0.943	0.953	−0.010
Period 4	0.857	0.919	−0.062
Period 5	0.929	0.900	0.029
Endline	0.957	0.991	−0.033**
Overall	0.924	0.938	−0.014

Notes: This table reports response rates for phone surveys (PS1–PS5) and the endline survey as well as overall. The control group has 70 participants, while the treated group has 320 participants.

Statistical significance at the 0.10, 0.05, and 0.01 levels is indicated by *, **, and ***.

**Table A.2:** Association between alternate mental health measures and work offer acceptance.

	(1)	(2)	(3)
Willingness to work outside			
High PHQ-2	−0.039		
	(0.029)		
High GAD-2		−0.049*	
		(0.029)	
High PHQ-2 and GAD-2			−0.078**
			(0.035)
Dep. Var. Mean	0.89	0.89	0.89
Willingness to work from home			
High PHQ-2	0.002		
	(0.022)		
High GAD-2		0.027	
		(0.021)	
High PHQ-2 and GAD-2			0.003
			(0.024)
Dep. Var. Mean	0.95	0.95	0.95
p-value: W.f. Home == W. outside	0.10	0.0030	0.0072
Observations	491	491	491

*Note:* This table presents correlations between mental health indicators and willingness to work in two settings: working outside and working from home. “Willingness to work from home” and “Willingness to work outside” are binary indicators for whether the respondent accepted the respective work offers. High PHQ-2 and High GAD-2 are binary indicators equal to one if the respondent’s score on the Patient Health Questionnaire-2 and Generalized Anxiety Disorder-2 screening tools exceeds their respective clinical screening threshold. High PHQ-2 and GAD-2 indicates respondents who score above the threshold on both measures. The table presents two panels: acceptance of work from home offers and acceptance of work from site offers. The p-value in the last row tests the equality of coefficients between work from home and work outside specifications within each column. All specifications include controls for whether the work from site question was asked first (the order was randomized), whether both the household head and spouse were present, the age of the respondent, their marital status and the number of adults and number of household members in the household at the time of the survey. The robust standard errors are in parentheses.

Statistical significance at the 0.10, 0.05, and 0.01 levels is indicated by *, **, and ***.

**Table A.3:** Correlation between mental health and willingness to work (without additional controls).

	(1)	(2)	(3)
Willingness to work outside			
PHQ-2 (std)	−0.034**		
	(0.017)		
GAD-2 (std)		−0.034**	
		(0.016)	
Ave. of PHQ-2 (std) and GAD-2 (std)			−0.043**
			(0.019)
Dep. Var. Mean	0.89	0.89	0.89
Willingness to work from home			
PHQ-2 (std)	−0.000		
	(0.013)		
GAD-2 (std)		0.004	
		(0.012)	
Ave. of PHQ-2 (std) and GAD-2 (std)			0.003
			(0.015)
Dep. Var. Mean	0.95	0.95	0.95
p-value: W.f. Home == W. outside	0.010	0.0025	0.0014
Observations	491	491	491

*Note:* This table examines the relationship between mental health measures and willingness to work, distinguishing between work-from-home and work-from-site offers. “Willingness to work from home” and “Willingness to work outside” are binary indicators for whether the respondent accepted the respective work offers. The analysis uses the short-form mental health questionnaires - PHQ-2 (Patient Health Questionnaire-2 for depression) and GAD-2 (Generalized Anxiety Disorder-2 for anxiety) - with scores standardized (std). The “Ave. of PHQ-2 (std) and GAD-2 (std)” takes the mean of these two standardized variables. We control for the order of work from site and work from home questions, and whether both head and spouse were present. The robust standard errors are in parentheses.

Statistical significance at the 0.10, 0.05, and 0.01 levels is indicated by *, **, and ***.

**Table A.4:** Work offers, labor supply, income and alternate mental health measures.

	(1)	(2)	(3)	(4)
	–	High PHQ-8	High GAD-7	High PHQ-8 and GAD-7
Working				
Job offer	0.435***	0.466***	0.443***	0.447***
	(0.032)	(0.038)	(0.036)	(0.035)
Baseline mental health measure		0.111*	0.051	0.070
		(0.062)	(0.070)	(0.080)
Baseline mental health measure × job offer		−0.098	−0.038	−0.072
		(0.069)	(0.078)	(0.088)
Control Mean	0.172	0.172	0.172	0.172
Observations	2188	2188	2188	2188
Days worked				
Job offer	1.693***	1.781***	1.745***	1.739***
	(0.204)	(0.242)	(0.229)	(0.217)
Baseline mental health measure		0.431	0.298	0.473
		(0.399)	(0.459)	(0.567)
Baseline mental health measure × job offer		−0.292	−0.228	−0.321
		(0.436)	(0.491)	(0.598)
Control Mean	1.052	1.052	1.052	1.052
Observations	2188	2188	2188	2188
Bags produced				
Baseline mental health measure		−0.168	0.066	−0.056
		(0.337)	(0.332)	(0.392)
Mean		8.998	8.998	8.998
Observations		1920	1920	1920
Work income				
Job offer	103.232***	105.256***	105.278***	103.968***
	(3.559)	(4.485)	(3.716)	(3.872)
Baseline mental health measure		4.983	8.926	5.535
		(6.264	(9.142)	(9.441)
Baseline mental health measure × job offer		−6.050	−8.594	−4.722
		(7.298)	(9.904)	(10.366)
Control Mean	14.97	14.97	14.97	14.97
Observations	2188	2188	2188	2188

*Note:* This table reports regression coefficients examining the relationship between job offers, labor market outcomes, and binary mental health measures in the Phase 2 sample of 390 participants across 6 rounds of post-baseline data collection. “High PHQ-8” (Patient Health Questionnaire-8 for depression) and “High GAD-7” (Generalized Anxiety Disorder-7 for anxiety) indicate scores above the clinical diagnoses thresholds for major depression and generalized anxiety disorder, respectively. The analysis covers four key outcome variables: Working (binary indicator), Days Worked (count of days), Bags produced (count of bags in each period), and Work Income (combines study and non-study income, in local currency units). All regressions include period fixed effects, controls to indicate whether the Phase 2 participant was also the Phase 1 respondent or was referred by a female respondent (leaving being referred by a male as the omitted group), as well as demographic controls for age and marital status of the respondent, household size and number of adults in the household. The standard errors, clustered at the household level, are in parentheses.

Statistical significance at the 0.10, 0.05, and 0.01 levels is indicated by *, **, and ***.

**Table A.5:** Work offers, labor supply, income and short-form mental health measures.

	(1)	(2)	(3)	(4)
	–	PHQ-2 (std)	GAD-2 (std)	Ave. of PHQ-2 (std) and GAD-2 (std)
Working				
Job offer	0.435***	0.432***	0.431***	0.429***
	(0.032)	(0.032)	(0.033)	(0.033)
Baseline mental health measure		0.056**	0.017	0.049
		(0.028)	(0.029)	(0.034)
Baseline mental health measure × job offer		−0.033	0.010	−0.015
		(0.032)	(0.033)	(0.038)
Control Mean	0.172	0.172	0.172	0.172
Observations	2188	2188	2188	2188
Days worked				
Job offer	1.693***	1.679***	1.670***	1.664***
	(0.204)	(0.204)	(0.209)	(0.207)
Baseline mental health measure		0.253	0.141	0.263
		(0.181)	(0.201)	(0.230)
Baseline mental health measure × job offer		−0.103	−0.027	−0.080
		(0.203)	(0.221)	(0.254)
Control Mean	1.052	1.052	1.052	1.052
Observations	2188	2188	2188	2188
Bags produced				
Baseline mental health measure		0.245	0.133	0.258
		(0.173)	(0.152)	(0.186)
Mean		8.998	8.998	8.998
Observations		1920	1920	1920
Work income				
Job offer	103.232***	102.892***	102.747***	102.563***
	(3.559)	(3.546)	(3.779)	(3.703)
Baseline mental health measure		6.739*	3.253	6.668
		(4.023)	(4.364)	(5.202)
Baseline mental health measure × job offer		−3.811	−1.883	−3.713
		(4.429)	(4.718)	(5.597)
Control Mean	14.97	14.97	14.97	14.97
Observations	2188	2188	2188	2188

*Note:* This table reports regression coefficients examining the relationship between job offers, labor market outcomes, and standardized continuous mental health measures in the Phase 2 sample of 390 participants across 6 rounds of post-baseline data collection. The analysis uses the short-form mental health questionnaires - PHQ-2 (Patient Health Questionnaire-2 for depression) and GAD-2 (Generalized Anxiety Disorder-2 for anxiety) - with scores standardized (std) for consistency with Phase 1 results. The “Ave. of PHQ-2 (std) and GAD-2 (std)” takes the mean of these two standardized variables. The analysis covers four key outcome variables: Working (binary indicator), Days Worked (count of days), Bags produced (count of bags in each period), and Work Income (combines study and non-study income, in local currency units). All regressions include period fixed effects, controls to indicate whether the Phase 2 participant was also the Phase 1 respondent or was referred by a female respondent (leaving being referred by a male as the omitted group), as well as demographic controls for age and marital status of the respondent, household size and number of adults in the household. The standard errors, clustered at the household level, are in parentheses.

Statistical significance at the 0.10, 0.05, and 0.01 levels is indicated by *, **, and ***.

**Table A.6:** Work offers, labor supply, income and mental health measures (without additional controls).

	(1)	(2)	(3)	(4)
	–	PHQ-8 (std)	GAD-7 (std)	Ave. of PHQ-8 (std) and GAD-7 (std)
Working				
Job offer	0.436***	0.434***	0.431***	0.432***
	(0.032)	(0.032)	(0.032)	(0.032)
Baseline mental health measure		0.040	0.032	0.041
		(0.025)	(0.025)	(0.026)
Baseline mental health measure × job offer		−0.038	−0.014	−0.029
		(0.030)	(0.030)	(0.032)
Control Mean	0.172	0.172	0.172	0.172
Observations	2188	2188	2188	2188
Days worked				
Job offer	1.698***	1.687***	1.670***	1.675***
	(0.201)	(0.202)	(0.203)	(0.203)
Baseline mental health measure		0.178	0.197	0.214
		(0.188)	(0.173)	(0.195)
Baseline mental health measure × job offer		−0.165	−0.123	−0.162
		(0.208)	(0.193)	(0.217)
Control Mean	1.052	1.052	1.052	1.052
Observations	2188	2188	2188	2188
Bags produced				
Baseline mental health measure		−0.072	0.136	0.039
		(0.179)	(0.167)	(0.193)
Mean		8.998	8.998	8.998
Observations		1920	1920	1920
Work income				
Job offer	103.303***	103.188***	102.661***	102.861***
	(3.569)	(3.606)	(3.709)	(3.663)
Baseline mental health measure		4.581	5.115	5.550
		(3.916)	(4.054)	(4.315)
Baseline mental health measure × job offer		−5.726	−4.045	−5.592
		(4.409)	(4.494)	(4.846)
Control Mean	14.97	14.97	14.97	14.97
Observations	2188	2188	2188	2188

*Note:* This table examines the relationship between mental health and labor market outcomes in the Phase 2 sample of 390 participants across 6 rounds of post-baseline data collection. PHQ-8 (std) and GAD-7 (std) refer to standardized scores from the Patient Health Questionnaire-8 and Generalized Anxiety Disorder-7 screening tools. The “Ave. of PHQ-8 (std) and GAD-7 (std)” takes the mean of these two standardized variables. The table presents four panels: Working (binary indicator), Days Worked (count of days), Bags produced (count of bags in each period), and Work Income (combines study and non-study income, in local currency units). All regressions include period fixed effects but no other controls. The standard errors, clustered at the household level, are in parentheses.

Statistical significance at the 0.10, 0.05, and 0.01 levels is indicated by *, **, and ***.

## Appendix B. Spillovers of mental health conditions to other household members

### Overview.

In Phase 1, survey respondents reported both their own willingness to work and that of other household members. Here, we examine how the respondent’s mental health relates to their reports of other household members’ willingness to work.

### Sample.

In this analysis 337 female respondents reported on the willingness to work of 500 female family members. The number of respondents is smaller than in Phase 1 (n = 491) because some lived in households without other eligible females.^18^

### Empirical Approach.

To examine the association between mental health and the reported willingness to work of *other household members*, we adjust Eq. (1) and estimate:

(3)
$$Y_{i,j}=\beta_{0}+\beta_{1\;}\text{Mentel}\;{\text{health}}_{i}+\beta_{2}X_{i}+\zeta_{i,j}$$

where *Y*_*i,j*_ is a binary variable equal to one if respondent *i* says that individual *j* is willing to work and zero otherwise. The other variables remain as described previously and results are again disaggregated by work location in two panels. In this specification, standard errors are clustered at the respondent level, given that each respondent can report the willingness to work of several household members.

### Results.

Table B.1 presents the results of regressions of the reported willingness to work of individuals *other than the respondent* as reported by the respondent on the mental health of the respondent, using the same standardized mental health measures as in previous analyses. More depressed respondents are significantly less likely to report that others in their household would be willing to take up the work offered, whether that work occurs in the household or outside of it. These effects are large: each standard deviation increase in the average of the two mental health measures is associated with a 4.2 percentage point decrease in the reported likelihood that other household members would work outside the home. These effects are robust to using binary measures of mental health (Table B.2). Those who are neither anxious nor depressed refuse only 11% of the time, while those who are both anxious and depressed refuse an additional 8% of the time.

**Table B.1:** Association between mental health and work offer acceptance for others.

	(1)	(2)	(3)
Willingness to work outside			
PHQ-2 (std)	−0.035**		
	(0.015)		
GAD-2 (std)		−0.031**	
		(0.015)	
Ave. of PHQ-2 (std) and GAD-2 (std)			−0.042**
			(0.017)
Dep. Var. Mean	0.87	0.87	0.87
Willingness to work from home			
PHQ-2 (std)	−0.036**		
	(0.014)		
GAD-2 (std)		−0.008	
		(0.013)	
Ave. of PHQ-2 (std) and GAD-2 (std)			−0.028*
			(0.015)
Dep. Var. Mean	0.91	0.91	0.91
p-value: W.f. Home == W. outside	0.91	0.033	0.18
Observations	500	500	500

*Note:* This table examines the relationship between the mental health of 337 female respondents in the Phase 1 sample and their reports of the willingness to work of other female members of their households. “Willingness to work from home” and “Willingness to work outside” are binary indicators for whether the respondent reported that the household member would accept the respective work offer. PHQ-2 (std) and GAD-2 (std) refer to standardized scores from the respondent’s own Patient Health Questionnaire-2 and Generalized Anxiety Disorder-2 screening tools. The “Ave. of PHQ-2 (std) and GAD-2 (std)” takes the mean of these two standardized variables. The top panel shows how respondent’s mental health correlates with their assessment of other household members’ availability for work-from-home opportunities, while the bottom panel shows the same for work-from-site opportunities. All specifications include controls for whether the work from site question was asked first (the order was randomized), whether both the household head and spouse were present, the age of the respondent, their marital status and the number of adults and number of household members in the household at the time of the survey. The p-value in the last row tests the equality of coefficients between work from home and work outside within each column. Standard errors, clustered at the household level, are in parentheses.

Statistical significance at the 0.10, 0.05, and 0.01 levels is indicated by *, **, and ***.

**Table B.2:** Association between alternate mental health measures and work offer acceptance for others.

	(1)	(2)	(3)
Willingness to work outside			
High PHQ-2	−0.036		
	(0.030)		
High GAD-2		−0.059**	
		(0.030)	
High PHQ-2 and GAD-2			−0.073**
			(0.035)
Dep. Var. Mean	0.87	0.87	0.87
Willingness to work from home			
High PHQ-2	−0.054**		
	(0.027)		
High GAD-2		−0.026	
		(0.026)	
High PHQ-2 and GAD-2			−0.075**
			(0.031)
Dep. Var. Mean	0.91	0.91	0.91
p-value: W.f. Home == W. outside	0.38	0.11	0.95
Observations	500	500	500

*Note:* This table examines the relationship between the mental health of 337 female respondents in the Phase 1 sample and their reports of the willingness to work of other female members of their households. “Willingness to work from home” and “Willingness to work outside” are binary indicators for whether the respondent reported that the household member would accept the respective work offer. High PHQ-2 and High GAD-2 are binary indicators equal to one if the respondent’s score on the Patient Health Questionnaire-2 and the Generalized Anxiety Disorder-2 screening tools exceeds their respective clinical screening threshold. “High PHQ-2 and GAD-2” indicates scores above the threshold on both measures. The top panel shows how respondent’s mental health correlates with their assessment of other household members’ willingness to work from home, while the bottom panel shows the same for work-from-site opportunities. All specifications include controls for whether the work from site question was asked first (the order was randomized), whether both the household head and spouse were present, the age of the respondent, their marital status and the number of adults and number of household members in the household. The p-value in the last row tests the equality of coefficients between Work from Home and Work from Site specifications within each column. The standard errors, clustered at the household level, are in parentheses.

Statistical significance at the 0.10, 0.05, and 0.01 levels is indicated by *, **, and ***.

Many potential explanations for this pattern exist. Differences in intra-household dynamics or communication, cognitive biases, correlated shocks that worsen mental health across household members, or reporting issues could all play a role. Data limitations prevent us from fully disentangling these mechanisms. However, Table B.3 offers suggestive evidence for one potential channel: consistent with symptoms of depression such as lack of motivation, participants with poor mental health may be less willing to take on additional domestic duties, creating spillovers to other household members. When participants declined to sign up female household members, those with poor mental health were more likely to cite childcare responsibilities as the reason, even controlling for the number of young children present.

**Table B.3:** Association between depression and reasons for work refusal for others.

	(1)	(2)	(3)	(4)	(5)	(6)
	Have work	Household work	Unable or unwilling	Student	Transportation	Not sure if person is interested
PHQ-2 (std)	0.035	0.067	−0.007	0.014	−0.022	−0.052*
	(0.027)	(0.041)	(0.033)	(0.028)	(0.021)	(0.026)
GAD-2 (std)	−0.045*	0.077**	0.020	−0.002	0.045	−0.048
	(0.026)	(0.037)	(0.043)	(0.025)	(0.035)	(0.041)
Ave. of PHQ-2 (std) and GAD-2 (std)	−0.004	0.096**	0.008	0.008	0.016	−0.067
	(0.023)	(0.047)	(0.045)	(0.032)	(0.030)	(0.041)
Controlling for number of children						
PHQ-2 (std)	0.033	0.066	−0.006	0.015	−0.023	−0.051*
	(0.026)	(0.042)	(0.033)	(0.029)	(0.022)	(0.027)
Number of young children (12 yrs and younger)	0.064**	0.012	−0.029	−0.041	0.033	−0.036
	(0.031)	(0.048)	(0.061)	(0.030)	(0.022)	(0.030)
GAD-2 (std)	−0.050*	0.077*	0.023	0.001	0.043	−0.046
	(0.026)	(0.038)	(0.041)	(0.026)	(0.035)	(0.041)
Number of young children (12 yrs and younger)	0.070**	0.007	−0.031	−0.040	0.028	−0.033
	(0.032)	(0.050)	(0.060)	(0.030)	(0.020)	(0.029)
Ave. of PHQ-2 (std) and GAD-2 (std)	−0.008	0.096*	0.010	0.011	0.014	−0.065
	(0.024)	(0.048)	(0.043)	(0.032)	(0.030)	(0.041)
Number of young children (12 yrs and younger)	0.066**	0.008	−0.030	−0.041	0.031	−0.033
	(0.031)	(0.049)	(0.061)	(0.030)	(0.021)	(0.029)
Reason share	0.116	0.125	0.429	0.196	0.0462	0.0804
Observations	112	112	112	112	65	112

*Note:* This table examines how a female respondent’s mental health relates to their stated reasons for why other female household members would not accept either work-fromhome or work-from-site opportunities. PHQ-2 (std) and GAD-2 (std) refer to standardized scores from the respondent’s own Patient Health Questionnaire-2 and Generalized Anxiety Disorder-2 screening tools. The “Ave. of PHQ-2 (std) and GAD-2 (std)” takes the mean of these two standardized variables. “# children” is the number of household members who are 12 years old or younger. Each column represents a different reason for refusing either type of work opportunity: (1) already having work, (2) household work responsibilities, (3) being unable or unwilling to work, (4) being a student, (5) transportation difficulties (only relevant to the work outside), and (6) uncertainty about the person’s interest. All specifications include controls for whether the work from site question was asked first (the order was randomized), whether both the household head and spouse were present, the age of the respondent, their marital status and the number of adults and number of household members in the household at the time of the survey. Reason Share indicates the proportion of respondents who cited each reason for other household members’ work refusal. Standard errors, clustered at the household level, are in parentheses.

Statistical significance at the 0.10, 0.05, and 0.01 levels is indicated by *, **, and ***.

Given the complexity of intra-household dynamics and potential confounding factors, these results should be viewed as suggestive rather than conclusive. Nonetheless, they indicate that mental health conditions may affect not only individual labor supply but also household-level economic opportunities. Further, the patterns observed are consistent with findings from Lund et al. (2019) across six countries, although further research is needed to clarify the mechanisms underlying these associations.
